# A quality assurance phantom for electronic portal imaging devices

**DOI:** 10.1120/jacmp.v12i2.3350

**Published:** 2011-02-02

**Authors:** Indra J. Das, Minsong Cao, Chee‐Wai Cheng, Vladimir Misic, Klaus Scheuring, Edmund Schüle, Peter A.S. Johnstone

**Affiliations:** ^1^ Department of Radiation Oncology Indiana University School of Medicine Indianapolis IN USA; ^2^ Indiana University Health Proton Therapy Center Bloomington IN USA; ^3^ Department of Radiation Oncology University of Pittsburgh Medical Center Natrona Heights PA USA; ^4^ PTW‐Freiburg Freiburg Germany

**Keywords:** QA, EPID, imager, MTF

## Abstract

Electronic portal imaging device (EPID) plays an important role in radiation therapy portal imaging, geometric and dosimetric verification. Consistent image quality and stable radiation response is necessary for proper utilization that requires routine quality assurance (QA). A commercial ‘EPID QC’ phantom weighing 3.8 kg with a dimension of $25\times 25\times 4.8\;{\text{cm}}^{3}$ is used for EPID QA. This device has five essential tools to measure the geometric accuracy, signal‐to‐noise ratio (SNR), dose linearity, and the low‐ and the high‐contrast resolutions. It is aligned with beam divergence to measure the imaging and geometric parameters in both X and Y directions, and can be used as a baseline check for routine QA. The low‐contrast tool consists of a series of holes with various diameters and depths in an aluminum slab, very similar to the Las Vegas phantom. The high‐resolution contrast tool provides the modulation transfer function (MTF) in both the x‐ and y‐dimensions to measure the focal spot of linear accelerator that is important for imaging and small field dosimetry. The device is tested in different institutions with various amorphous silicon imagers including Elekta, Siemens and Varian units. Images of the QA phantom were acquired at 95.2 cm source‐skin‐distance (SSD) in the range 1–15 MU for a $26\times 26\;{\text{cm}}^{2}$ field and phantom surface is set normal to the beam direction when gantry is at 0° and 90°. The epidSoft is a software program provided with the EPID QA phantom for analysis of the data. The preliminary results using the phantom on the tested EPID showed very good low‐contrast resolution and high resolution, and an MTF (0.5) in the range of 0.3–0.4 lp/mm. All imagers also exhibit satisfactory geometric accuracy, dose linearity and SNR, and are independent of MU and spatial orientations. The epidSoft maintains an image analysis record and provides a graph of the temporal variations in imaging parameters. In conclusion, this device is simple to use and provides testing on basic and advanced imaging parameters for daily QA on any imager used in clinical practices.

PACS number: 87.57 C‐, 87.57 N‐

## I. INTRODUCTION

Advances in imaging have revolutionized the delineation of target volume and organs at risk for treatment planning in radiation oncology. To verify patient positioning, digitally reconstructed radiographs (DRR)^(^ ^1^ ^–^ ^3^ ^)^ from a treatment plan are usually compared with portal images either in analog forms (radiographs) or in digital format with an electronic portal imaging device (EPID).^(^ ^4^ ^–^ ^6^ ^)^ Changes in EPID technology are providing unsurpassed image quality for patient setup and verification. The reviews of EPID technology have been presented by several investigators for older and outdated systems.^(^ ^4^ ^–^ ^7^ ^)^ Modern linear accelerators are equipped with advanced EPID designs which utilize amorphous silicon technology (also known as flat panels) and have superior image quality compared to the older devices based on video camera or ion chamber technology. EPID has become an essential component in high‐precision radiation therapy providing digital images that can be enhanced to aid visualization and interpretation. Image verification is subject to accuracy of the data acquired and, to some extent, on the post‐processing algorithms. Modern radiation therapy has become image‐based, where the role of EPID has expanded from patient setup and portal verification^(^ ^8^ ^–^ ^10^ ^)^ to more comprehensive uses such as MLC QA,^(11)^ localization of implantable markers in IGRT,^(12)^ IMRT QA^(^ ^13^ ^–^ ^16^ ^)^ and absolute dosimetry.^(^ ^17^ ^–^ ^19^ ^)^

Each machine vendor provides their own proprietary post‐processing software with the EPID to help visualize the treatment field manually or automatically. To successfully perform these tasks, routine QA of an EPID has been recommended.^(^ ^4^ ^,^ ^7^ ^,^ ^20^ ^–^ ^22^ ^)^ Unfortunately, most of these reports discuss the necessity of the QA and use of an in‐house phantom for the individual institutional use, but is lacking in widespread availability and intercomparison data. Lack of a suitable phantom for EPID QA could compromise the goal of precision therapy in IGRT or IMRT. Early approaches of visual evaluation of portal images are subjective and qualitative. However, the accuracy required in imaging and dosimetry in modern radiation therapy demands consistent image quality and quantitative tests that are robust, precise, accurate and automatic.

A preliminary evaluation of the QC phantom has been reported by Das et al.^(23)^ A similar study by Pesznyák et al.^(24)^ has reported multiple difficulties in the image processing of the associated imaging software, mainly gray scale reversal (black to white and vice versa), after exporting images from some portal imagers. When gray scale reversal took place, the signal‐to‐noise ratio (SNR) was incorrectly recorded with increasing absorption, which may pose a problem in routine QA. The revised software version 2.2 has eliminated all of the image processing difficulties. In this study, an evaluation of the updated PTW EPID phantom and QC software (PTW‐Freiburg, Freiburg, Germany) is investigated for Elekta iView, Siemens BeamView Plus and Varian aSi1000 EPID systems from three different institutions under various clinical conditions. The goal of this study is not to quantify the absolute performance parameters, but rather the QA of imaging devices across several platforms in a clinical setting, and also to provide a baseline data and temporal patterns very similar to the one from a daily QA device for machine output.

## II. MATERIALS AND METHODS

A short description of the EPID system used in this study has been provided in various references^(^ ^5^ ^,^ ^6^ ^,^ ^25^ ^,^ ^26^ ^)^ and are highlighted here. An Elekta iView amorphous silicon EPID (Elekta Ltd, Crawley, UK) with a detector panel from Perkin‐Elmer (Fremont, CA, USA) is used. The active area of detector is $41\times 41\;{\text{cm}}^{2}$ consisting of a copper plate and a $133{\text{mg}/\text{cm}}^{-2}$ terbium‐doped gadoliniumoxysulfide $\left({\text{Gd}}_{2}O_{2}S:\text{Tb}\right)$ screen. The distance from the source to the sensitive layer of the detector is 160 cm. The maximum area that could be imaged at the isocenter is $25.6\times 25.6\;{\text{cm}}^{2}$. There are 1024×1024 pixels in the image. (Additional details of the Elekta EPID system are provided in the references.^(^ ^6^ ^,^ ^25^ ^)^) Siemens BeamView Plus EPID system (Siemens AG, Munich, Germany) uses Perkin Elmer flat panel with a 0.6 mm aluminum plate and ${\text{Gd}}_{2}O_{2}S:\text{Tb}$ phosphor screen. The pixel format is 1024×1024 with a pixel pitch of 400 μm. The array dimension is $41\times 41\;{\text{cm}}^{2}$ and with a source‐to‐detector distance of 140 cm, resulting in a field of view at isocenter of just $29\times 29\;{\text{cm}}^{2}$. (Additional information of this system can be found in the references^(^ ^5^ ^,^ ^26^ ^)^.) The Varian aSi1000 amorphous silicon EPID system (Varian Medical Systems, Palo Alto, CA) is a flat‐panel indirect detector based on ${\text{Gd}}_{2}O_{2}S:\text{Tb}$ detector with dimensions of $30\times 40\;{\text{cm}}^{2}$ with an array of 768×1024 photodiodes giving an effective pixel size of 390 μm at a source to detector distance of 150 cm.^(^ ^6^ ^,^ ^18^ ^,^ ^19^ ^,^ ^27^ ^,^ ^28^ ^)^ Based on the manufacturer's specification, Elekta, Siemens and Varian EPIDs have 2.0, 2.5, 2.56 pixel/mm giving a Nyquist frequency (fn) of 1.0, 1.25, 1.28 lp/mm, respectively.

### A. EPID QC Phantom

**Figure 1:**
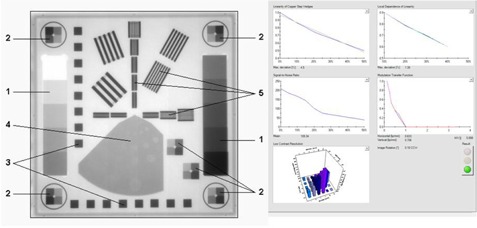
Arrangement of test elements in PTW EPID QC phantom: (1) signal linearity and signal‐to‐noise ratio; (2) isotropy of signal linearity; (3) geometric isotropy (distortion); (4) low‐contrast resolution; (5) high‐contrast resolution. Also shown are sample analyses in the epidSoft user interface.

The EPID QC phantom marketed by PTW‐Freiburg (Germany) was developed specifically for checking the consistency of the image quality of EPID for high‐energy X‐rays in radiation therapy. The associated software epidSoft (version 2.2) provided with the QC phantom allows automatic image analysis of the QC phantom images, documentation of the QC results, and data storage and retrieval for long‐term assessment of the performance of the EPID. The outer dimensions of the QC phantom are $25\times 25\times 4.8\;{\text{cm}}^{3}$ and weighs 3.8 kg. The device provides analysis of $26\times 26\;{\text{cm}}^{2}$ field size at 95.2 cm SSD that is covered by most imagers at their source to imager distances. The unique feature of this device is the collection of imaging tools for EPID QA including basic aspects such as: 1) geometric evaluation including scaling and rotation tests, 2) linearity test of absorption through a pair of copper step wedges (5% for 6 MV), 3) local dependence of linearity, 4) signal‐to‐noise ratio (SNR), 5) low‐contrast resolution through various holes in aluminum slab, and 6) high‐contrast spatial resolutions through MTF determination in both the in‐plane and cross‐plane directions since resolution depends on the focal spot which varies in both directions. The arrangement of the test elements in the QC phantom is shown in Fig. 1. A brief description of each test element and its calculation method is given below.

## B. GEOMETRIC TEST

When an EPID image of the QC phantom is first loaded into epidSoft, the position of the QC phantom is calibrated by defining four corner points of the image. It is done by clicking the center of the four individual elements at the corners, which are used to test signal linearity (Fig. 1) by means of the magnifying glass tool. When the corner points are defined, all test elements will be automatically identified and marked with blue frames. Any rotation of the QA phantom position will be calculated and displayed.

### C. Linearity test of absorption through Cu steps

The two copper step wedges are used for linearity determination. Each wedge consists of five steps and together they cover the range of 0% to 50% absorption (0%, 5%, 10%, 15%, 20%, 25%, 30%, 35%, 40% and 50%) for a 6 MV beam. The linearity curve is calculated from the mean gray values of the 10 steps. For the display, an additional 45% value is calculated from the 40% and 50% values by linear interpolation, which gives a total of 11 data points. A regression line is then calculated from these 11 points. The maximum deviation between the measured and the regressed values is then calculated based on the goodness of the fit.

#### C.1 Local dependence of linearity

The local dependence of linearity is determined by the brass steps in the corners and two additional ones in the third quadrant of the phantom (Fig. 1). Each of the brass steps consists of four steps which cover approximately 10%, 20%, 30% and 40% absorption for 6 MV X‐rays. A linearity curve is calculated for each block from the mean gray values of the steps. A regression line is generated from the mean values of the four steps and the output value is calculated as the maximum deviation between the measured value and the regressed one.

#### C.2 Signal‐to‐noise ratio (SNR)

The signal‐to‐noise ratio (SNR) is also determined by means of the copper steps. The SNR is calculated for each absorption level of the copper step by the following formulas:

(1) $$SNR=\frac{\bar{N(x,y)}}{\sqrt{\sigma^{2}}}$$

(2) $$\sigma^{2}=\int_{F}[N{\left(x,y\right)}^{2}-\bar{N{\left(x,y\right)}^{2}}]dF$$

(3) F=4xy; x ε{−X,X}; y ε{−Y,Y}

where $\sigma^{2}$ is the variance of the gray values, $\bar{N(x,y)}$ is the arithmetic mean gray value of an area F (the region of interest), *N(x, y)* is the gray value at position (x, y) and *X* and *Y* are the specific coordinate values. The output mean value is then calculated as the average of SNR values of the ten copper steps.

#### C.3 Low‐contrast resolution through various holes

The low‐contrast resolution is determined by the test element with 27 holes of different diameters (range from 1.1–15 mm) and depths (0.5–4.8 mm). These holes are arranged in a 6 by 5 array (six columns and five rows), with holes in each row having the same diameter, while holes in each column have the same depth. For each hole, the contrast difference of the hole and a specified area around the hole is calculated and displayed in tabular and graphic forms.

#### C.4 High‐contrast and spatial resolutions

The line pattern in the first and second quadrants of the phantom are used for the determination of the modulation transfer function (MTF) and the high‐contrast resolution in the in‐plane and cross‐plane directions. There are 14 lamella blocks with 18 resolutions in a range of 0.167 lp/mm to 3.5 lp/mm. Four blocks are arranged diagonally for simultaneous determination of the in‐plane and cross‐plane MTF. The remaining ten blocks are divided into two groups of five, each arranged in in‐plane and cross‐plane directions respectively. The outer block of each group contains two different resolutions. The mean gray values of the lamellae (maxima) and the mean gray values of the gaps (minima) are determined for each lamella block. The luminance density distribution g(x) at a location x of an ideal image can be described in a sinusoidal form:

(4) g(x)=1+k⋅sin(2⋅π⋅ν⋅x)

where *x* is the spatial location, υ is the spatial frequency of the grid, 1/υ is the distance between two neighboring maxima, *k* is the amplitude (0≤k ≤ 1). Each line of the grid contributes to the same image. The image b(x') of the object g(x) is represented in terms of the line spread function (LSF) as:

(5) $$b(x^{\prime })=\int_{-\infty }^{\infty }g(x)\cdot L(x^{\prime }-x)dx$$

and can be displaced by converting and introducing a phase shift by phase angle ϕ as:

(6) $$b(x^{\prime })=1-\eta \cdot k\cdot \sin(2\cdot \pi \cdot \nu \cdot x^{\prime }-\phi )$$

The difference between object g(x) and image b(x') is a change of amplitude from η to kη and a phase shift ϕ between image and object grid, respectively. The function η(υ) then becomes MTF(υ) as:

(7) η=MTF(ν)

The MTF is calculated as the ratio of contrast k, of the object grid g(x) to image grid b(x'):

(8) $$k=\frac{\max(g(x))-\min(g(x))}{\max(g(x))+\min(g(x))}$$

(9) $$k\eta =\frac{\max(b(x^{\prime }))-\min(b(x^{\prime }))}{\max(b(x^{\prime }))+\min(b(x^{\prime }))}$$

(10) $$MTF(\nu )=\left(\frac{\max(b(x^{\prime }))-\min(b(x^{\prime }))}{\max(b(x^{\prime }))+\min(b(x^{\prime }))}\right)|\left(\frac{\max(g(x))-\min(g(x))}{\max(g(x))+\min(g(x))}\right)$$

The MTF(υ) is calculated based on image contrast, gray values of lamella (maximum) and intermediate space (minimum) and object using Eq. 10. The details of this method is given by Coleman.^(29)^ The calculated MTF values are then normalized to the smallest available spatial frequency to generate a relative MTF.

### D. Image quality check

The EPID QC phantom was used for commercially available EPID devices on Elekta, Siemens and Varian linear accelerators in this study. These EPID devices are of the latest design with superior image quality based on amorphous silicon technology. To perform the quality tests of each imager, the QC phantom was placed on the treatment couch at a location free of any material absorption by placing it on the tennis racket of the treatment couch with the front plate facing the beam. The gantry angle was set to be precisely perpendicular to the phantom. The source‐to‐imager distance was set in such a way that all test elements can be seen completely in the EPID image. The field size was set to 26 cm×26 cm at the isocenter so that it covers all test elements of the phantom completely. Due to the focus geometry of the phantom, it is placed at 95.2 cm source‐to‐surface distance to provide the base at isocenter. This can be simply achieved by aligning the recessed lines on the side of the phantom (horizontal and vertical direction) with the laser. This device was tested with 6 MV beams on an Elekta iView, a Siemens BeamView Plus and a Varian aSi1000 EPID system, respectively. Images were acquired with 1–15 MU exposures and phantom surface set to be normal to the beam direction and gantry set to 0° and 90°, respectively. The dose or dose rate was chosen depending on the EPID (100 MU/min on the Varian Linac, 100 MU/min for Elekta, and 50 MU/min on the Siemens), so that no overexposure or underexposure is visible in the EPID images and all copper levels should be visible and the resolution should be optimal. Images were analyzed with epidSoft and a report is generated at completion of the analysis. The epidSoft also allows preset values to be compared with periodic evaluation of these parameters and plots the summary and trend over time.

## III. RESULTS

An image of the phantom with the Varian aSi1000 EPID imager displayed in the epidSoft user interface is shown in Fig. 1. It shows a complete pattern of all test tools in one unit and the analyzed results of one exposure. The robustness of the phantom and epidSoft is obvious, as all relevant imager performance characteristics are displayed in one screen for easy evaluation. For example, in addition to the linearity and MTF, the low‐contrast resolution is shown as a 3D histogram (Fig. 1) in the format of six columns and five rows, which corresponds to the spatial arrangement of all test boreholes in the phantom. The histogram can be expanded and rotated to evaluate the relative contrast difference of each specific borehole.

**Figure 2:**
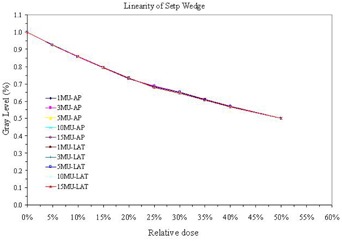
Linearity of step wedge measured with 1–15 MU and different orientation (AP vs. LAT) for a Siemens imager. Note that MU and orientation cannot be differentiated and, hence, any orientation or MU could be used.

**Figure 3:**
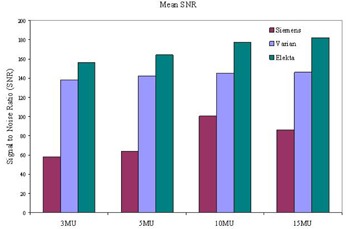
Normalized mean SNR values at different MUs for three imagers. It indicates that low MU could be used for the QA for all imagers.

**Figure 4:**
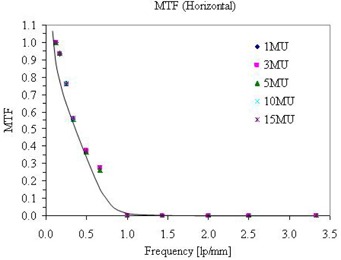
MTF of a Varian imager in in‐plane direction measured by different MUs.

As shown in Fig. 2, the Varian aSi1000 exhibits linearity over the range 1–15 MU, for the AP and LAT beams. The mean SNR increased slightly with MUs, as shown in Fig. 3. It was observed that imaging orientation (AP and Lat) was not a factor in the QA outcome. Usually MTF is difficult to determine for therapy imaging devices, as noted in the literature for DRR.^(^ ^1^ ^,^ ^30^ ^)^ However, the PTW QC phantom provides a seamless MTF analysis in both the in‐plane and cross‐plane directions. The goal of this study is not to determine the absolute MTF or the accuracy of MTF, but rather to provide a baseline and temporal shift in the EPID performance. Figure 4 shows the MTF in the in‐plane direction of a Varian aSi1000 imager acquired with different MUs. As expected, the MTF measurement is also independent of MU used. A better representation of the MTF for such data is to plot on a logarithmic scale and include data only up to the Nyquist frequency of the EPID. A request has been made to the vendor for modification in the software upgrade.

**Figure 5:**
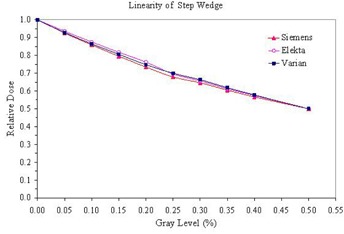
Comparison of linearity measurement of step wedge from three different EPID: Elekta iView, Siemens BeamView Plus and Varian aSi1000 imager.

**Figure 6:**
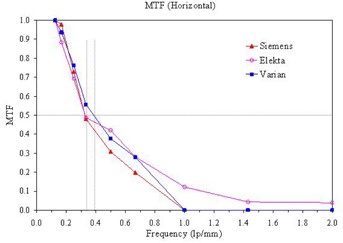
MTF in in‐plane direction measured from Elekta iView, Siemens BeamView Plus and Varian aSi1000 imagers. Based on specifications, the Nyquist frequency of Elekta, Siemens and Varian imagers are 1.0, 1.25, 1.28 lp/mm, respectively.

Comparing different commercially available EPID devices, most of them provide similar data of linearity and spatial resolution. Figure 5 shows measurements of linearity of the step wedges for the three different EPID devices used in this study. The three devices provide very close measurement at each grey level and there is no significant difference in the overall linearity. The MTF curves in the in‐plane direction measured from these EPID devices are also compared in Figure 6. The MTFs of Siemens and Varian imagers are very similar, while the MTF of the Elekta device shows slightly higher values at high frequency region. Overall, these three devices provide similar values of MTF (0.5) in the range of 0.3–0.4lp/mm, which agree with those reported earlier by various investigators.^(^ ^4^ ^,^ ^7^ ^)^

**Figure 7:**
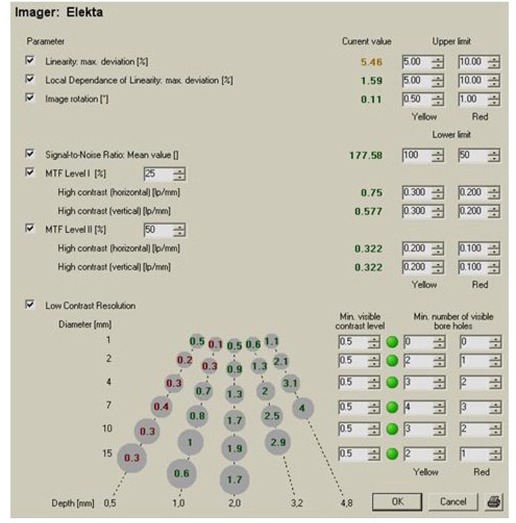
Analysis pattern of one of the data for an imager, showing all QA parameters and their preset values, as well as tolerances. A user can use the preset values to compare the data on a periodic basis.

**Figure 8:**
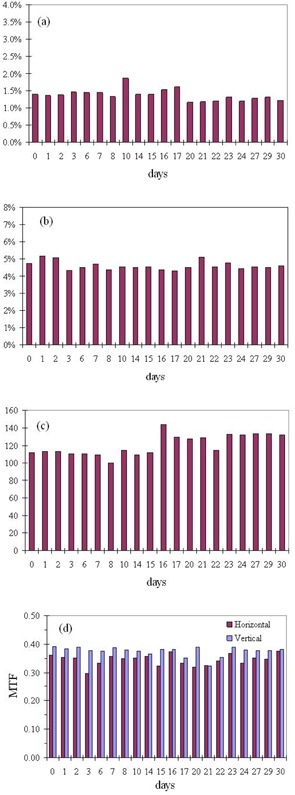
Critical QA parameters (a) local linearity, (b) linearity, (c) SNR and (d) MTF are automatically plotted in the statistical mode of the epidSoft for temporal analysis graphically.

An important feature of the epidSoft is that it allows preset values of QA parameters to be set as baselines for periodic evaluation of the imaging device. Figure 7 shows the user interface where the values of all QA parameters can be set as baselines. The software maintains an individual database for each EPID device for analysis of the pattern of variation on a weekly, monthly and yearly basis. Figure 8 shows the temporal QA analysis of a Varian imager over one month. It was observed that no significant change has occurred during this period of time. Such observations are important for the other two imagers (Elekta iView and Siemens BeamView Plus); however, the temporal data for these imagers is not available since the device was loaned to us for a limited period of time.

## IV. DISCUSSION

Since its introduction in radiation therapy in the early 1980s, electronic portal imaging has played a very important role in treatment verification and other clinical applications. Successful implementation of an EPID in clinical radiation oncology requires careful and regular quality assurance and maintenance of the performance of EPID devices. However, according to a survey conducted by AAPM Task Group 58 (TG58),^(4)^ only 20% of the institutions with EPID have developed a comprehensive QA program, and fewer than half of them perform the QA program regularly. About 35% of the institutions with EPID do not have any QA program. A possible reason is the lack of appropriate QA device and appropriate software for data analysis. A common QA phantom called Las Vegas phantom has been developed and used in acceptance testing and routine QA for EPID image quality evaluation.^(4)^ It is composed of holes with different depths and diameters, and is embedded in an aluminum block which can be used to check spatial and contrast resolution of EPID device. However, the evaluation process is based on visual inspection of the phantom image which is relatively subjective and time‐consuming. Compared with Las Vegas phantom, the analysis process of the QA phantom used in this paper is highly automated and quantitative. If the device is lined up perpendicular to the central axis of the beam, the image of the four corner points could be defined by the user using a magnification tool. The software (epidSoft) automatically calculates all imaging parameters, providing an objective evaluation very efficiently. Another advantage of this QC phantom is that it contains a set of comprehensive test components. Thus in one exposure, it allows simultaneous evaluation of all imaging parameters as recommended by AAPM TG‐58,^(4)^ such as SNR, resolution and linearity. The whole process only takes 3–5 minutes. The AAPM TG‐58 recommended a daily check of imaging functionality and a monthly check of image quality. Our QA results acquired over a one‐month period did not show any significant change of the image quality parameters. Thus it is not unreasonable to imbed the image quality check as part of the monthly linac QA. On the other hand, since the entire QA procedure using this EPID QA phantom is highly automated and only takes a few minutes, it might be a good practice to perform the QA tests and analysis using the EPID QA phantom on a daily basis. The QA image acquired not only serves as image functionality check, but also can be quickly compared with baseline values to prevent sudden change in the imager performance due to unexpected mechanical or electronic malfunction. Another important test recommended by AAPM TG‐58, which is not included in the design of this EPID phantom, is the geometric localization accuracy. However, the center of the four corner elements on the phantom can be used as reference points for geometric measurement accuracy test since the distance between the four elements are known and can be used to compare with measured distance.

The concept of automatic recognition of test components and calculation of QA parameters used in this EPID QA system provided a solid base for the development of QA phantoms and software for other imaging systems that are widely used in radiation therapy, such as kV based imager and cone‐beam CT. The calculation algorithm in epidSoft may be simply adapted to kV imager and cone‐beam CT systems with some modifications so that automatic image quality QA and analysis can be performed. This will definitely improve the efficiency and accuracy of current QA procedures for these imaging systems.

The system, however, has a few limitations as described below.

1. The software currently supports all popular image format (DICOM, BMP, TIFF), but images still need to be manually imported into epidSoft. An automatic connection to vendor imaging acquisition system will streamline the procedure.
2. The phantom size is too big for the Varian image detector. The detector has to be moved up very close to isocenter, which is not a common clinical position.
3. The baseline QA parameters are left to the therapy physicists to decide. A simple guideline based on expert opinion should be provided as a default.

## V. CONCLUSIONS

The PTW EPID QC phantom with associated image analysis software provides a convenient, automated and easy process for periodic verification of performance of an EPID with regard to baseline values. This device is independent of EPID system and provides tools for continuous monitoring of the various parameters, such as geometrical accuracy, exposure linearity, SNR, and low‐contrast resolution and high‐contrast resolution (MTF). Such a device is ideal for image evaluation and for QA when EPID is used for dosimetry.
